# Experienced and Perceived Risks of Mycobacterial Diseases: A Cross Sectional Study among Agropastoral Communities in Northern Tanzania

**DOI:** 10.1371/journal.pone.0130180

**Published:** 2015-06-24

**Authors:** Andrew Martin Kilale, Esther Ngadaya, Gibson Benard Kagaruki, Yakobo Leonard Lema, Julius Muhumuza, Bernard James Ngowi, Sayoki Godfrey Mfinanga, Sven Gudmund Hinderaker

**Affiliations:** 1 National Institute for Medical Research, Muhimbili Centre, Dar es Salaam, Tanzania; 2 National Institute for Medical Research, Tukuyu Centre, Tukuyu, Mbeya, Tanzania; 3 Centre for International Health, University of Bergen, Bergen, Norway; Public Health Research Institute at RBHS, UNITED STATES

## Abstract

**Objective:**

The current study was conducted to assess experienced risk factors and perceptions of mycobacterial diseases in communities in northern Tanzania.

**Methods:**

We conducted a cross-sectional study in Arusha and Manyara regions in Northern Tanzania. We enrolled tuberculosis (TB) patients attending Mount Meru Hospital, Enduleni Hospital and Haydom Lutheran Hospitals in Arusha municipality, Ngorongoro and Mbulu districts, respectively. Patient addresses were recorded during their first visit to the hospitals. Patients with confirmed diagnosis of TB by sputum smear microscopy and/or culture at central laboratory were followed up and interviewed using pre-tested questionnaires, and selected relatives and neighbors were also interviewed. The study was conducted between June 2011 and May 2013.

**Results:**

The study involved 164 respondents: 41(25%) were TB patients, 68(41.5%) were their relatives and 55(33.5%) their neighbors. Sixty four (39%) knew a risk factor for mycobacterial disease. Overall, 64(39%) perceived to be at risk of mycobacterial diseases. Exposure to potential risks of mycobacterial diseases were: keeping livestock, not boiling drinking water, large family, smoking and sharing dwelling with TB patients. Rural dwellers were more often livestock keepers (p<0.01), more often shared dwelling with livestock (p<0.01) than urban dwellers. More primary school leavers reported sharing dwelling with TB patients than participants with secondary and higher education (p = 0.01).

**Conclusion:**

Livestock keeping, sharing dwelling with livestock, sharing household with a TB patient were perceived risk factors for mycobacterial diseases and the participants were exposed to some of these risk factors. Improving knowledge about the risk factors may protect them from these serious diseases.

## Background

Mycobacterial infection is a global public health problem in humans, animal populations and ecosystems, particularly in developing countries [1,2]. *Mycobacterium tuberculosis (MTB)* is the principal cause of human tuberculosis worldwide [3], but environmental mycobacteria (non-tuberculous mycobacteria, NTM) are opportunistic pathogens whose role in human and animal disease is increasingly recognized [4]. *Mycobacterium bovis* and a wide range of species of NTM have been isolated and characterized from environment shared by humans and animals [3, 5, 6]. Many mycobacterial infections are preventable and the associated diseases are treatable with multiple special antibiotics for a long duration [7].

In the last few decades, the incidence of classical tuberculosis in humans has been on the increase alongside tuberculosis-like disease caused by environmental mycobacteria [8,9]. Tanzania ranks fourteenth among TB high burdened countries in the world [2]. A total of 65,732 cases of all forms were notified in 2013, which shows an increase of 1,840 cases or 2.9 percent compared to the year 2012. The notification rate of tuberculosis (all forms) in 2013 remained at 142 cases per 100,000 populations as for the year 2012. Notification rate of new smear positive tuberculosis cases decreased from 56 to 53 cases per 100,000. This however, shows that TB is still a major burden in the country [10]. In the HIV/AIDS era, mycobacterial infections have become a subject of interest because of their potential co-infection with HIV/AIDS [11]. It has been estimated that one-third of HIV/AIDS deaths are due to mycobacterial diseases, particularly tuberculosis [12].

The human-mycobacterial interaction with its impact on human health is complex and likely broader than currently recognized [1]. Human activities are likely to influence the distribution and prevalence of mycobacteria. The role of open water sources with its livestock/wildlife as reservoirs of infections to human are well documented [11,13]. In agropastoral ecosystems with close interaction between humans, livestock and wildlife affects the health of humans. These semi-arid areas in general suffer from poor quality pastures and seasonal water availability [4]. In such areas, large herds of cattle, domestic animals such as goats and sheep, wildlife, and humans all share the same environment, providing opportunities for close interaction and potential risk of mycobacterial infection to these communities [12].

Effective prevention and control measures of mycobacterial diseases require comprehensive initiatives that address the primary barriers for the prevention of mycobacterial infection. In this regard it is important to understand the perceptions that influence behavior and response to mycobacterial diseases. Knowledge about risk factors for TB has been studied in Tanzania, but information on potential risk factors of mycobacterial diseases in general for individuals, families and communities in the most at-risk areas of Tanzania is limited. This study aims to study selected socio-cultural risk factors and knowledge about them in this area. Our specific objectives were to assess the following among TB patients, their relatives and neighbors in three selected districts with many agropastoralists in Tanzania: 1) their knowledge about some known risk factors; 2) their exposure to these risk factors; 3) the association between exposure to risk and their risk perception.

## Methods

### Design

We conducted a cross-sectional study to assess knowledge and exposure to risks of mycobacterial infections among TB patients, their relatives and neighbors.

### Setting

The study was conducted in the catchment areas of 3 hospitals in Northern Tanzania: Mount Meru Hospital in Arusha municipal, Enduleni Catholic Hospital in Ngorongoro district and in Haydom Lutheran Hospital in Mbulu district. The TB patients were enrolled from these hospitals. According to the 2012 Census the selected districts had a population of 650,370 [14]. The notification rate of all types of TB in Arusha and Manyara regions in 2011 were between 150–199 per 100 000 population [15]. Prominent local tribes include the Maasai in Ngorongoro, the Arusha and the Meru in Arusha, and the Datoga and the Iraqw in Mbulu district, and many homes in all these groups are agropastoralists.

### Sampling of the study subjects

This study was part of a larger study where a total of 1,711 eligible TB suspects attending the three selected health facilities were enrolled for a study on diagnostics of mycobacterial infections. Sputum samples from the enrolled participants were processed and examined for smear microscopy at the local health facility to allow case management; this information was not used in this study. At the same time, another sample from the enrolled patients was collected and send to the Central Tuberculosis Reference Laboratory (CTRL) in Dar es Salaam for smear microscopy culture and drug sensitivity testing was also done for all positive isolates. From the 1,711 TB suspects, there were 277 proven TB patients from whom we selected participants. The inclusion criteria for TB patients in this study were: 1) smear or culture positive samples; 2) residents of the study area; 3) consenting to participate; 4) traceable by mobile phone and address recorded at the health facility. Using patient addresses recorded at the health facility during the initial visit, the patients were listed for follow up and interviews to assess their experiences and perception on mycobacterial diseases. Prior to visits for interview patients were called by phone and consent was requested. For each consenting TB patient, relatives who were present and neighbors living in the vicinity were also interviewed.

### Data collection

Data was collected using two structured questionnaires: one for patients and one for relatives and neighbors. The questionnaires included standard questions adapted from previous studies [16] and some developed based on existing literature [17]. The questionnaires were translated into Swahili (the national language) and pre-tested for clarity and cultural acceptability, and the information collected was as follows: Baseline information including sex, age, area of residence, marital status, occupation and level of education; perceptions on selected risk factors for mycobacterial infections, like number of people sharing a dwelling, history of sharing house with a person with TB, past history of TB infection, smoking and livestock keeping, consumption of raw animal products, household primary source of water, source of drinking water for animals/livestock, and preparation of drinking water for the family. Some known risk situations assessed in this study are listed in Table 1 [12,18,19]. Eligible TB patients were enrolled between June 2011 and May 2012, relatives and neighbors between 2012 and 2013.

**Table 1:** Situations associated with risk of mycobacterial diseases (references in brackets).

Livestock keeping	[3,4]
Never boil, filter or treat drinking water	[4]
Large family ≥5 individuals	[4]
Smoker or ex-smoker	[4,18]
Sharing dwelling with a TB patient	[4]
Sharing water source with animals	[4]
Sharing dwelling with livestock	[4]
Sleeping 4≥ individuals in one room	[4]
Consume raw milk	[12,13]
Consume raw meat	[18,19]
Consume raw blood	[4]

### Ethical considerations

Ethics approval was given by the National Health Research Ethics Review Committee of the National Institute for Medical Research in Tanzania. Both regional and district administrative officials gave permission to conduct the study in their areas. All respondents were asked to sign a consent form prior to participation in the study.

### Data management and analysis

All responses in the study questionnaires were coded and double entered in Epi-data version 3.1. Data was transferred to Statistical Package for Social Sciences version 18 for windows (SPSS Inc, Chicago, USA) for analysis. Pearson Chi square statistics test was used to compare categorical variables. Significance level was set at p<0.05.

## Results

In Table 2 we present a summary of the socio-demographic characteristics of the study participants. The study involved 164 respondents of whom 41(25%) were confirmed TB patients and 68(41.5%) were their relatives and 55(33.5%) their neighbors. The mean age of the study participants was 39 (range 14–76) years.

**Table 2:** Socio-demographic characteristics of study participants by study group in northern Tanzania, 2011–12.

Characteristic	Total	TB patients	Relatives	Neighbors
	n (%)	n (%)	n (%)	n (%)
**Sex**				
Male	84 (51)	24 (58)	28 (41)	32 (58)
Female	80 (49)	17 (42)	40 (59)	23 (42)
**Age group**				
≤20	14 (9)	4 (10)	7 (10)	3 (6)
21–30	39 (24)	6 (15)	17 (25)	16 (29)
31–40	46 (28)	12 (29)	19 (28)	15 (27)
41–50	26 (16)	3 (7)	14 (21)	9 (16)
>50	39 (24)	16 (39)	11 (16)	12 (22)
**Marital status**				
Married	119 (73)	26 (63)	52 (77)	41 (75)
Others (single, widow)	45 (27)	15 (37)	16 (23)	14 (25)
**Region**				
Arusha	59 (36)	19 (46)	25 (37)	15 (27)
Manyara	105 (64)	22 (54)	43 (63)	40 (73)
**District**				
Mbulu	106 (65)	22 (54)	43 (63)	41 (75)
Ngorongoro	18 (11)	2 (5)	10 (15)	6 (11)
Arusha	40 (24)	17 (41)	15 (22)	8 (14)
**Level of education**				
No formal education	38 (23)	13 (32)	19 (28)	6 (11)
Primary	99 (60)	19 (46)	44 (65)	36 (65)
Secondary and above	27 (17)	9 (22)	5 (7)	13 (24)
**Residence**				
Urban	47 (29)	20 (49)	17 (25)	10 (18)
Rural	117 (71)	21 (51)	51 (75)	45 (82)
**Occupation**				
Peasant	117 (71)	23 (56)	53 (78)	41 (75)
Employed	13 (8)	2 (5)	4 (6)	7 (13)
Business	23 (14)	10 (24)	8 (12)	5 (9)
None	11 (7)	6 (15)	3 (4)	2 (4)

### Knowledge about some known risk factors

Table 3 shows the responses by the three groups of participants to questions about risk factors for mycobacterial diseases. Overall, 61% of the 164 respondents reported that they did not know any risk factor for mycobacterial diseases: 49% of TB patients, 59% of relatives and 73% of neighbors. Living with a person who had TB, sharing eating and drinking utensils, and having infectious diseases were risk factors known for the infections.

**Table 3:** Knowledge about some known risk factors for mycobacterial diseases in northern Tanzania, 2011–12.

Response	Total	Patients	Relatives	Neighbors
	n (%)	n (%)	n (%)	n (%)
All respondents	164 (100)	41 (100)	68 (100)	55 (100)
Yes, I know a risk factor	64 (39)	21 (51)	28 (41)	15 (27)
No, I don’t know risk factors	100 (61)	20 (49)	40 (59)	40 (73)
**Selected known risk factors**				
Living with a person with TB	20(12)	6(15)	9(13)	5(9)
Sharing eating and drinking utensils	11(7)	5(12)	6(9)	-
Infectious diseases can transmit	11(7)	4(10)	5(7)	2(4)
Having ever suffered from TB	7(4)	3(7)	3(4)	1(2)
Inhaling infected air from coughing person	7(4)	2(5)	3(4)	2(4)
Coughing or fevers	2(1)	-	1(1)	1(2)
Dirty environment	2(1)	-	-	2(4)
Attending public events	1(1)	-	1(1)	-
Witchcraft	1(1)	1(2)	-	-
Congestion	1(1)	-	-	1(2)

### Perception of being at risk from mycobacterial diseases

Overall, 64(39%) of the 164 study participants perceived to be at risk from mycobacterial diseases; 51% of the TB patients, 41% of the relatives and 27% of the neighbors. In Table 4 we present the respondents’ perceptions of being at risk from mycobacterial diseases by demographic characteristics. A smaller fraction of the neighbors than the patients felt at risk. Compared to the age group over 50 years, a smaller proportion of those aged 21–30 years felt at risk. Respondent’s marital status, level of education, residence and occupation were not significantly associated with perception of being at risk from mycobacterial diseases.

**Table 4:** Perceptions of being at risk of mycobacterial diseases, by demographic characteristics in northern Tanzania, 2011–12.

Characteristic		Consider being at risk?		
	Total	Yes, n (%)	No, n (%)	Risk Ratio and 95% CI
**Sex**				
Male	84	34(41)	50(60)	1.09; (95%CI 0.735–1.584)
Female	80	30(38)	50(63)	
**Age group**				
< = 20	14	5(36)	9(64)	0.66; (95%CI 0.310–1.419)
21–30	39	8(21)	31(80)	0.38; (95%CI 0.193–0.754)
31–40	46	16(35)	30(65)	0.65; (95%CI 0.395–1.055)
41–50	26	14(54)	12(46)	1.00; (95%CI 0.632–1.583)
>50	39	21(54)	18(46)	
**Marital status**				
Married	119	49(41)	70(59)	1.24; (95%CI 0.775–1.968)
Others (single, widow, widower)	45	15(33)	30(67)	
**Region**				
Arusha	59	21(51)	38(64)	0.87; (95%CI 0.575, 1.314)
Manyara	105	43(41)	62(59)	
**District**				
Mbulu	106	43(41)	63(59)	
Ngorongoro	18	3(17)	15(83)	0.41; (95%CI 0.143–1.184)
Arusha	40	18(45)	22(55)	1.11; (95%CI 0.734–1.676)
**Type respondent**				
Patients	41	21(51)	20(49)	
Relative	68	28(41)	40(59)	0.80; (95%CI 0.532–1.214)
Neighbor	55	15(27)	40(73)	0.53; (95%CI 0.315–0.900)
**Level of education**				
No formal education	38	17(45)	21(55)	1.34; (95%CI 0.708–2.545)
Primary	99	38(38)	61(62)	1.15; (95%CI 0.639–2.075)
Secondary and above	27	9(33)	18(67)	
**Residence**				
Urban	47	18(38)	29(62)	0.97; (95%CI 0.636–1.493)
Rural	117	46(39)	71(67)	
**Occupation**				
Peasant	117	47(40)	70(60)	
Employed	13	4(31)	9(69)	0.77; (95%CI 0.329–1.783)
Business	23	10(44)	13(57)	1.08; (95%CI 0.646–1.813)
None	11	3(27)	8(73)	0.68; (95%CI 0.252–1.827)

### Association between perceived risk and exposure to potential risks of mycobacteria

We asked the participants about eleven practices with some inherent risk of exposure to mycobacteria, listed in Table 1. In Table 5 we display some of these common situations with risk of exposure to mycobacteria and the reported perceptions of risks for mycobacterial diseases by the study respondents. Respondents who do not boil, filter or treat their drinking water considered themselves to be at risk of mycobacterial diseases (p = 0.05); so also did the respondents who had shared dwelling with a known TB patient (p<0.01). Participants who had shared dwelling with TB patients felt more at risk (p<0.01) as did livestock keepers (p<0.01) and those who never boil or treat their drinking water (p = 0.05). Other associations were not statistically significant. Rural dwellers were more often livestock keepers and more exposed to this potential source of infection (p<0.01), and they shared dwelling with livestock more frequently than urban dwellers (p<0.01). Males and females had similar exposures to situations with potential exposure, although more males consumed raw meat (14: 23%) than females (4: 8%, p = 0.05). Patients and relatives and neighbors had similar exposure. There were more livestock keepers among participants in Mbulu (67; 41%) than in Ngorongoro (10; 6%) and Arusha districts (11; 7%), (p<0.01). A higher proportion of participants who had (at least started) primary school education had shared dwelling with TB patients than those without any schooling (p = 0.01).

**Table 5:** Exposure to situations with known potential risk of mycobacterial diseases in northern Tanzania, 2011–12.

Exposure	Responded	Consider being at risk?	
		Yes, n (%)	No, n (%)
Livestock keeping	88	51 (58)	37 (42)
Never boil, filter or treat drinking water	77	47 (61)	30 (39)
Large family ≥5 individuals	75	45 (60)	30 (40)
Smoker or ex-smoker	71	40 (56)	31(44)
Sharing dwelling with a TB patient	67	46 (69)	21 (31)
Sharing water source with animals	49	26 (53)	23 (47)
Sharing dwelling with livestock	37	23 (62)	14 (38)
Sleeping 4≥ individuals in one room	21	14 (67)	7 (33)
Consume raw milk	26	14 (54)	12 (46)
Consume raw meat	18	10 (56)	8 (44)
Consume raw blood	16	7 (44)	9 (56)

## Discussion

Studies focusing on individual experience on situations and perceived risks of mycobacterial diseases in rural and urban agropastoral communities in Tanzania are limited. The current study shows that livestock keeping, sharing dwelling with livestock, sharing household with a TB patient, living in congested households and consumption of raw meat were the main potential exposures for mycobacterial diseases in the study area. Similarly, rural residence, being a male, a peasant and holding a primary school education were significant socio-demographic characteristics associated with exposure to risky practices. The study shows that sixty one percent of the respondents did not perceive that they were at risk of being infected with mycobacteria.

Previous studies have shown that individuals who perceive to be at risk of contracting a disease are likely to take measures to protect themselves against the disease [20]. Sixty percent of our study participants consider themselves not at risk, and were therefore not likely to take any measures for protection from mycobacterial diseases. Overcrowding is a known risk factor for TB [21–23], and we found that almost half of the participants were living in houses with many persons; however, less than 15% shared sleeping room with four or more.

Despite evidence indicating that cultural and socio-economic factors, among others, increases the likelihood of mycobacterial transmission, consumption of raw animal products including raw milk, meat and drinking untreated water poses a risk of transmitting mycobacteria to humans [20]. Earlier reports associate the consumption of raw milk and meat with extra-pulmonary TB [24–26], and Ameni and colleagues [27] reported no association between consumption of raw milk and occurrence of human TB. In agropastoral communities milk is often produced and consumed at household level as opposed to milk pooled for a wider commercial purpose which is pasteurized. Therefore milk has minimal role in transmitting mycobacteria to people other than the household.

We showed that among TB patients the perception of risk and exposure to it was statistically associated for people sharing dwelling with TB patients and livestock keepers, whilst other risk factors were not. This underlines the fact that knowledge about risk does not in itself create a protected environment, as many other circumstances play a role. Furthermore, in our analysis the relation between the timing of the exposure and the perception is not known. Measures for protection from TB patients were still in place in hospital isolation wards during the initial weeks of treatment.

Although awareness does not necessarily translate into behavior, these findings have public health implications calling for appropriate community interventions to reduce the risk of mycobacterial diseases in agropastoral communities in Tanzania and similar settings. Such interventions include health education to improve awareness, perceptions and change in practice. For the interventions to be effective, both the community and health system has to address the complex socio-cultural aspects surrounding the agropastoralists.

Our study has several limitations. Firstly; despite the reported findings, not all the 277 proven TB patients were enrolled into the study as many did not fulfill inclusion criteria. The small sample size limited the analysis we could carry out from our research questions. Secondly; the presence of mycobacterial pathogens in the environment was investigated in another study, but was not part of this study. Thirdly; as part of a larger study on mycobacteria, this resulted into low sample size for some of the outcomes.

## Conclusion

Our study shows that two third of the participants did not know a risk factor for mycobacterial disease. Livestock keeping, sharing dwelling with livestock, sharing household with a TB patient, congestion and consumption of raw meat were perceived risk factors for mycobacterial diseases. The participants were exposed to some of these risk factors. Exposure to these common potential risks was substantial but perception about the risk was limited. Improving knowledge about the risk factors may protect them from these serious diseases.

## References

[1] Primm TP, Lucero CA and Falkinham JO. Health Impacts of Environmental Mycobacteria. Clin. Microbiol. Rev. 2004; 17(1): 98–106. 14726457 10.1128/CMR.17.1.98-106.2004 PMC321467

[2] WHO. Global Tuberculosis Report 2013. WHO/HTM/TB/2013.15.

[3] Mfinanga SG, Mørkve O, Kazwala RR. Tribal differences in perception of tuberculosis: a possible role in tuberculosis control in Arusha, Tanzania. Int J Tuberc Lung Dis. 2003; 10: 933–941. 14552562

[4] Kankya C, Muwonge A, Olet S, Munyeme M, Biffa D, Opuda-Asibo J, et al Factors associated with pastoral community knowledge and occurrence of mycobacterial infections in Human-Animal Interface areas of Nakasongola and Mubende districts, Uganda. BMC Public Health. 2010; 10: 471 10.1186/1471-2458-10-471 20698978 PMC2924292

[5] Mfinanga SG, Morkve O, Kazwala RR, Cleaveland S, Sharp MJ, Kunda J, et al Mycobacterial adenitis: role of *Mycobacterium bovis*, non-tuberculous mycobacteria, HIV infection, and risk factors in Arusha, Tanzania. East Afr Med J. 2004; 81(4): 171–178. 15884281 10.4314/eamj.v81i4.9150

[6] Kankya C, Muwonge A, Djonne B, Munyeme M, Opuda-Asibo J, Skjerve E, et al Isolation of non-tuberculous mycobacteria from pastoral ecosystems of Uganda: Public Health significance. BMC Public Health. 2011; 11: 320 10.1186/1471-2458-11-320 21575226 PMC3123205

[7] Bryan C. Mycobacterial diseases. In: *Microbiology and Immunology On-line*, Hunt, R.C. editor. 2011. Available: http://www.microbiologybook.org/Infectious%20Disease/mycobacterial%20diseases.htm.

[8] Mawak J, Gomwalk N, Bello C, Kandakai-Olukemi Y. Human pulmonary infections with bovine and environment (atypical) mycobacteria in Jos, Nigeria. Ghana Med J. 2006; 40(4): 132–6. 17496986 10.4314/gmj.v40i3.55268 PMC1868006

[9] Corti M, Palmero D, Eiguchi K. Respiratory infections in immunocompromised patients. Curr Opin Pulm Med. 2009; 15(3): 209–217. 10.1097/MCP.0b013e328329bd2c 19276812

[10] National TB and Leprosy Programme. Ministry of Health and Social Welfare. National Tuberculosis and Leprosy 2013 Annual Report. NTLP Annual Report 2013.

[11] van Ingen J, Boeree MJ, Dekhuijzen PNR and Van Soolingen D. Environmental sources of rapid growing non-tuberculous mycobacteria causing disease in humans. Clin Microbiol Infect. 2009; 15: 888–893. 10.1111/j.1469-0691.2009.03013.x 19845700

[12] Oloya J, Opuda-Asibo J, Kazwala R, Demelash AB, Skjerve E, Lund A, et al Mycobacteria causing human cervical lymphadenitis in pastoral communities in the Karamoja region of Uganda. Epidemiol Infect. 2008; 136: 636–643. 17599779 10.1017/S0950268807009004 PMC2870852

[13] Ameni G, Vordermeier M, Firdessa R, Aseffa A, Hewinson G, Gordon SV, et al *Mycobacterium tuberculosis* infection in grazing cattle in central Ethiopia. Vet J. 2011; 188: 359–361. 10.1016/j.tvjl.2010.05.005 20965132 PMC3103825

[14] National Bureau of Statistics (Tanzania). Tanzania Population and Housing Census 2002. Available: http://ghdx.healthdata.org/record/tanzania-population-and-housing-census-2002. Accessed 2 June 2014.

[15] National TB and Leprosy Programme. Ministry of Health and Social Welfare. National Tuberculosis and Leprosy 2011 Annual Report. NTLP Annual Report 2011.

[16] Joseph HA, Waldman K, Rawls C, Wilce M, Shrestha-Kuwahara R. TB perspectives among a sample of Mexicans in the United States: results from an ethnographic study. J Immigr Minor Health. 2008; 10(2): 177–185. 17557205 10.1007/s10903-007-9067-5

[17] Shrestha-Kuwahara R and Joseph H. Perceptions of tuberculosis among foreign-born persons: an Ethnographic Study. Find TB Resources. 2006. Available: https://findtbresources.cdc.gov/popup/pop_08.htm. Accessed 2 May 2015.

[18] den Boon S, van Lill SWP, Borgdorff MW, Verver S, Bateman ED, Lombard CJ, Lombard CJ, et al Association between smoking and tuberculosis infection: a population survey in a high tuberculosis incidence area. Thorax. 2005; 60: 555–557. 15994262 10.1136/thx.2004.030924 PMC1747449

[19] Eltholth MM, Marsh VR, Van Winden S and Guitian FJ. Contamination of food products with *Mycobacterium avium paratuberculosis*: a systematic review. Journal of Applied Microbiology. 2009; 107: 1061–1071. 10.1111/j.1365-2672.2009.04286.x 19486426

[20] Malama S, Muma JB and Godfroid J. A review of tuberculosis at the wildlife-livestock-human interface in Zambia. Infectious Diseases of poverty. 2013; 2: 13 10.1186/2049-9957-2-13 23849550 PMC3734204

[21] Wanyeki I, Olson S, Brassard P, Menzies D, Ross N, Behr M, et al Dwellings, crowding, and tuberculosis in Montreal. Soc Sci Med. 2006; 63: 501–11. 16480805 10.1016/j.socscimed.2005.12.015

[22] Young D and Strasser S. An ecological view of the risk factors for tuberculosis in the United States. Journal of Public Health and Epidemiology. 2012; 4(1): 24–29.

[23] Siddiqui MS, Fakih HAM, Burney WA, Iftikhar R, Khan N. Environmental and host-related factors predisposing to tuberculosis in Karachi: A cross-sectional study. J Pak Med Stud. 2011; 1(1).

[24] Shitaye JE, Tsegaye W, Pavlik I.Bovine tuberculosis infection in animal and human population in Ethiopia: a review. Vet Med. 2007; 8:317–332.

[25] Legesse M, Ameni G, Mamo G, Medhin G, Shawel D, Bjune G, et al Knowledge and perception of pulmonary tuberculosis in pastoral communities in the middle and Lower Awash Valley of Afar region, Ethiopia. BMC Public Health. 2010; 10: 187 10.1186/1471-2458-10-187 20380747 PMC2867998

[26] Munyeme M, Muma JB, Munang'andu HM, Kankya C, Skjerve E, Tryland M. Cattle owners' awareness of bovine tuberculosis in high and low prevalence settings of the wildlife-livestock interface areas in Zambia. BMC Vet Res. 2010; 6(21): 1–9. 10.1186/1746-6148-6-21 20406464 PMC2874791

[27] Ameni G, Amenu K, Tibbo M. Bovine tuberculosis: prevalence and risk factor assessment in cattle and cattle owners in Wuchale-Jida district, Central Ethiopia. Int J Appl Res Vet Med. 2003; 1(1): 1–13.

